# Characterization of *Staphylococcus aureus* Strains Isolated from Veterinary Hospital

**DOI:** 10.1155/2020/2893027

**Published:** 2020-08-01

**Authors:** Ariel E. Stella, Thaís F. Lima, Cecília N. Moreira, Eric M. N. De Paula

**Affiliations:** ^1^Universidade Federal de Jataí—UFJ, Jataí, Goiás State, Brazil; ^2^Unifimes, Mineiros, Goiás State, Brazil

## Abstract

This study aims to detect *Staphylococcus aureus* (*S. aureus*) resistance in the veterinary hospital environment. *S. aureus* are one of the components of the microbiota, and they may be present in patients in a veterinary hospital environment. Methicillin resistance is determined by a chromosomal gene (*mecA*), which codes for modifications in the beta-lactam antibiotic receptor, where the penicillin-binding protein will have a low affinity for the antibiotic. Samples were collected through swabs of materials and equipment at the hospital. *S. aureus* was identified in 7.6% (21/276) of the samples collected, and of the 21 strains isolated, 4 (19.0%) carried the *mecA* gene. MRSA are all strains of *S. aureus* that express the *mecA* gene. Four strains harbor the *mecA* gene; however, only two expressed the phenotypic resistance to cefoxitin and were characterized as MRSA. An isolate (strain 18) present on a patient care table was identified as methicillin-resistant *S. aureus* with intermediate sensitivity to vancomycin (VISA). Our observations suggest the need for containment measures (good antisepsis practices) to avoid the possible transmission of resistant bacterial agents for the veterinary hospital environment.

## 1. Introduction

*Staphylococcus* sp. represents one of the main groups of microorganisms involved in human or animal infections. Penicillin and its derivatives, including methicillin, have been used for the treatments of infections caused by this microorganism; however, certain strains developed resistance known as Methicillin-Resistant Coagulase-Negative Staphylococci (MRS) and methicillin-resistant *S. aureus* (MRSA). This resistance to methicillin is determined by the *mecA* gene, which encodes the low affinity penicillin-binding protein PBP 2 [1]. The prevalence of MRSA/MRS strains in infections has increased worldwide, and an alternative treatment has been the use of na antibiotic glycopeptides such as vancomycin. However, it has emerged as another resistant profile of these microorganisms, the methicillin-resistant *S. aureus* with intermediate sensitivity to vancomycin (VISA). These bacteria may be disseminated to the community through colonized medical staff or discharged patients. The emergence and spread of MRSA in the community, independent of the healthcare setting and in the absence of typical risk factors for nosocomial MRSA infections, are matters of further concern [2]. An additional potential risk is born by emergence and spread and transferable antibiotic resistance genes coding, so far, unknown resistance mechanisms and accumulation of MRSA in animals, and transfer to humans, therefore, also has an impact on regulations of antibiotic usage in animals [3]. Dogs, cats, and horses have become an important part of most families; therefore, there are high chances of human colonization or infection with MRSA from these animals [4]. Therefore, there is a high chance of hospital colonization which may be from contact with an MRSA colonized patient or contaminated objects [1]. Also, the areas contaminated in the hospital include medical instruments, beddings, clothing, furniture, toiletries, and the atmosphere [5]. Since 2006, MRSA is widely disseminated among various livestock animals mainly as an asymptomatic nasal colonizer [6]. It can be introduced to hospitals and cause nosocomial infections such as postoperative surgical site infections, ventilator-associated pneumonia, septicemia, and infections after joint replacement. Because of its capacity to cause a variety of infections in humans, MRSA became a public health issue. Prevention of further dissemination of MRSA with a zoonotic potential needs concerted action of veterinary infection control specialists and clinicians [3, 7], but it is necessary to obtain information regarding the prevalence of MRSA infection before implementing strategies for infection control in veterinary medical practice. The data presented in the present study could provide some information regarding the transmission of MRSA/VISA in a veterinary hospital environment.

## 2. Materials and Methods

### 2.1. Bacterial Strains and Phenotypic Identification

Samples were collected at the Veterinary Hospital of UFG (Jataí-GO) as follows: swabs of desks, surgical tables, hospitalization cages, stethoscopes, thermometers, and any instruments used in direct contact with animals. Swabs were wetted in Butterfield's solution containing 0.1% of Tween 20 [8]. The collections were performed 9 times in periods not less than 30 days between each collection. The collected samples were cultured in nutrient broth, blood agar, and mannitol agar at 35°C for 24–48 hs. For the identification of *S. aureus*, the following tests were used: the Gram stain, catalase test, furazolidone and bacitracin sensitivity, coagulase test, DNAse test, mannitol fermentation, mannose fermentation, raffinose, sucrose, trehalose and xylose, maltose fermentation, and the VP test. Then, the pure cultures were stored with freezed glycerin at −80°C for further analysis.

### 2.2. Determination of the Antimicrobial Sensitivity of the Isolates

The methicillin-resistant *S. aureus* (MRSA) was investigated, and the method used was described by CLSI [9]. The minimal inhibitory concentration (MIC) test for vancomycin, clindamycin, and gentamicin antimicrobials was performed using the concentration of 0.016–256 *μ*g/mL present in the strips (Liofilchem), whereas for ciprofloxacin, the concentration was 0.002–32 *μ*g/mL and for sulfamethoxazole, the concentration was 0.064–1024 *μ*g/mL. Control strains included *S. aureus* ATCC 29213.

### 2.3. mecA-PCR

Bacterial DNA was obtained as follows: the strains were cultured in BHI broth for 12 hours at 35°C and, then, centrifuged at 5000 rpm for 4 min. The supernatant was discarded, and the pellet washed 3 times with 200 *μ*l of TE buffer. Subsequently, the pellet was resuspended in 100 *μ*l of TE buffer, and the microtubes were heated at 95°C in a water bath for 10 min and, then, centrifuged at 5000 rpm for 20 sec. The supernatant (100 *μ*L) was transferred to a microtube, frozen at −20°C, and stocked. The detection of the *mecA* gene by the polymerase chain reaction (PCR) was performed according Gortel et al. [10] with modifications established by Neves et al. [11] The primers used for the detection of a 533 bp fragment were 5′-AAA ATC GAT GGT AAA GGT TGG C 3′ and 5′ AGT TCT GCA GTA CCG GAT TTG C 3′. 2 *μ*L of the DNA diluted previously (30 ng); 2.5 *μ*l of 1x PCR buffer (50 mM KCl, 200 mM TRIS-HCl, pH 8.4); 2.5 U of Taq DNA polymerase; 0.2 mM dNTP; 1.5 mM MgCl2, 1 *μ*g of each primer, and sterile milli water were used until the reaction volume was 20 *μ*L. The samples were placed in a thermocycler with the following cycle: 2 min at 94°C; 1 min at 94°C; 2 min at 52°C; 2 min at 72°C; 39 cycles from step 2; 5 min at 72°C, and maintenance of the samples under refrigeration at 5°C. All products after the amplification process were analyzed on agarose gel with 1.5% ethidium bromide and processed at 65 V for 1 h 30 min.

## 3. Results

Of the 276 samples collected, 58 strains of coagulase-negative *Staphylococcus* (CoNS) (19%) and 21 *S. aureus* (6.8%) were isolated from the dog clipper, ultrasound table, stethoscope, telephone, consultation tables, door handle, dog muzzle, thermometer, and surgical instruments. Four strains of CoNS were resistant to cefoxitin, thus identified as MRS (1.45%), and two strains of *S. aureus* were resistant and identified as MRSA (0.64%). MRSA strains were isolated from the treatment table and from nonautoclaved surgical material, whereas MRS strains were isolated from the cage, muzzle, and nonautoclaved surgical material (Table 1). Four strains harbor the *mecA* gene; however, only two expressed the phenotypic resistance to cefoxitin and were characterized as MRSA (Table 1). No strain was resistant to vancomycin; however, an isolate (strain 18) present on a patient care table was identified as methicillin-resistant *S. aureus* with intermediate sensitivity to vancomycin (VISA). Most strains of *S. aureus* were not drug resistant (NDR); however, three strains were resistant to one or more classes of antibiotics being classified as single drug resistant (SDR).

## 4. Discussion

Both in human and veterinary medicine, there is a concern with MRSA, which has been gradually increasing due to the difficulty of combating them, being a worldwide public health problem where most of the infections occur through some lineages with pathogenic potential. MRSA are becoming increasingly frequent in nosocomial infections, both in the human environment and in the veterinary environment. In addition, reports of animals and humans serving as reservoirs and transporting bacteria to both environments [12] have been reported. In our study, we found a prevalence of 1.6% for MRS and 0.64% for MRSA, and it is observed that depending on the study site and sample size, the global rates are extremely variable (0.03% to 80%) [1]. Professionals who work in veterinary hospitals and, therefore, have frequent contact with animals should be trained on the risks of transmitting MRS and MRSA in the environment. In this work, two methicillin-resistant strains (15-MRSA and 24-MRS) were found in the surgical materials, and this evidences the importance of the correct autoclaving of this material because the possibility of transmission to animals (at a vulnerable time, such as surgery) is real and with disastrous consequences. In these clinics and hospitals, hygienic measures such as hand washing before and after contact with contaminated surfaces and avoiding close contact with the discharges from the nose, mouth, and wounds of infected humans and animals should be adopted. Our data demonstrated that MRSA and MRS strains may be circulating in the veterinary hospital setting, evidencing the significant contamination capacity of this microorganism, as well as its persistence in the environment. Knowing that the isolates of samples collected and found were cages, muzzles, nonautoclaved materials, desks, and stethoscopes; therefore, there is concern regarding nosocomial infections that have a connection with MRS and MRSA, since they are direct contact materials and between animals and the professionals who work there. In addition, the spatial distribution of MRSA may indicate interspecies transmission and colonization of different populations. Although hospital cleaning can reduce MRSA/MRS contamination in the environment, in some cases, it does not eliminate it. Veterinarians should be encouraged to choose antibiotics for therapy according to antibiogram tests, as well as to wear masks and gloves when handling patients.

In the case of vancomycin, which was first released in the 1950s, resistance was not reported until the mid 1990s, with the description of vancomycin intermediate-resistant *S. aureus* (VISA) [13]. Vancomycin is an antibiotic used for the treatment of Gram-positive bacterial infections. Traditionally, it has been used as a drug of last resort; however, clinical isolates of MRSA strains with decreased susceptibility to vancomycin, VISA, and more recently, with high-level vancomycin resistance (vancomycin-resistant *S. aureus* [VRSA]) have been described in the clinical literature [14]. In this study, an isolate (strain 18) was identified as methicillin-resistant *S. aureus* with intermediate sensitivity to vancomycin (VISA). An MRSA isolate with decreased susceptibility to vancomycin was first reported in Japan in 1997 [15]. As in our study, the isolated strain in Japan had only a modestly increased minimum inhibitory concentration (MIC) value for vancomycin, in the range of 3–8 *μ*g/ml. However, there have been reports of VISA strains with a vancomycin MIC value greater than 100 *μ*g/ml in the United States [13]. Therefore, in the case of these strains producing an infection, the use of this antibiotic, the last available option, is therapeutically impossible. VISA isolates do not carry imported foreign genetic elements; rather, the increased vancomycin MIC values are related to mutations that appear in the invading pathogen during vancomycin therapy in vivo. VISA began to be reported with increasing frequency among MRSA isolates identified all over the world [16].

Resistance to the other antibiotics tested was detected mainly in strains 5, 10, and 14, which were resistant to antibiotics of different classes, being classified as SDR. Resistance to drugs of different classes is worrying because it shows that bacteria are constantly passing through the process of genetic recombination and, consequently, they are acquiring endogenous genes, which, in the future, would allow the appearance of multidrug-resistant, extensively-resistant, or even pandrug-resistant strains. High-level resistance to methicillin is caused by the *mecA* gene, which encodes an alternative penicillin-binding protein, PBP 2a [17]. Interestingly, of the four strains carrying the *mecA* gene, two (5 and 7) did not phenotypically express this resistance, and this may be due to differences in the regulation of gene expression in these cells or to a lower sensitivity of the test. Additionally, strain 5, which carries the *mecA* gene, has been shown to be resistant to clindamycin and gentamicin, so we have here a potentially multidrug-resistant strain because in the case of expression of this gene, this bacterium will be resistant to three different classes of antibiotics [18]. In addition, the wide distribution of microorganisms through the environment facilitates the possibility of colonization and/or infection of a wide variety of hosts (humans and animals), and this allows the contact of strains of different genetic profiles, an essential factor for genetic recombination between them. In this sense, these bacteria can acquire genetic material related to mechanisms of resistance to antibiotics, which may be carried by pathogenic or nonpathogenic strains.

The clinical use of antimicrobials plays a role in the selection of resistant strains and is probably the main cause of resistance, especially in veterinary hospital where there is a higher concentration of antibiotic residues in the environment, as well as in the animal organism. As more bacterial strains become resistant to an increasing number of antibiotics, therapeutic options become increasingly limited and expensive and in some cases nonexistent. Effective interventions in the hospital environment are, therefore, necessary to minimize the problem of microbial resistance, the control of antimicrobial use and the control and prevention of hospital infections being the main interventions that can be performed in this sense. However, the resistance of the bacterial species to antimicrobials is extremely variable among countries and, in this sense, it is necessary that actions to control this situation and the definition of microorganisms to be monitored are planned based on global and local epidemiological information.

For hospital infection, the hands and nostrils of colonized individuals are the major sources of MRSA transmission. MRSA is released into the hospital environment either through aerosol, skin cells, or stools of an infected patient [19]. Gehanno et al. [20] found a similar strain of MRSA in patients of a hospital and the room atmosphere. Although hospital cleaning reduces MRSA contamination of the environment, in some cases, it does not eliminate it. The emergence of resistant microorganisms to the various classes of antimicrobials has been progressive in the last decades, constituting a threat to the world public health. The circulation of these strains (MRSA, MRS, and VISA) in the veterinary hospital environment is worrisome because the World Health Organization (WHO) classifies this profile as a high priority according to its list of pathogens according to the severity of the infections they cause and the number of antibiotics available for treatment. Our observations suggest the need for containment measures (good antisepsis practices) to avoid the possible transmission of resistant bacterial agents in the veterinary hospital environment.

## 5. Conclusions

MRS and MRSA strains were identified in the veterinary hospital environment. The presence of MRS and MRSA strains, in the veterinary hospital environment and instruments, indicates the possibility of circulation of these strains between the environment, animals, and humans. An isolate (strain 18) present on a patient care table was identified as methicillin-resistant *S. aureus* with intermediate sensitivity to vancomycin (VISA).

## Figures and Tables

**Table 1:** Pathotypes and minimum inhibitory concentration (MIC) of coagulase-negative *Staphylococci* (CoNS) and *S. aureus*.

Isolates	Microorganism	Antibiotic (*μ*g/ml)					*mecA*	Methicillin resistant	Resistance profile
		VAN	CLIN	GEN	CIP	Source			
1	*S. aureus*	0.75	0.125	0.023	0.38	Dog clipper			NDR
2	*S. aureus*	0.75	0.125	0.50	0.19	Ultrasound table			NDR
3	*S. aureus*	1.0	0.047	0.047	0.125	Stethoscope			NDR
4	*S. aureus*	0.094	0.75	0.047	0.25	Telephone			NDR
5	*S. aureus*	1.5	48^*∗*^	4^*∗*^	0.5	Consultation tables		•	MDR
6	*S. aureus*	1	0.19	0.19	0.064	Door handle			NDR
7	*S. aureus*	0.75	0.032	0.047	0.064	Door handle		•	SDR
8	*S. aureus*	0.25	0.064	0.023	0.047	Consultation tables		NDR	
9	*S. aureus*	0.75	1	0.016	0.19	Consultation tables			NDR
10	*S. aureus*	1	0.75	8^*∗*^	32^*∗*^	Dog muzzle			SDR
11	*S. aureus*	0.25	0.064	0.094	0.094	Ultrasound table			NDR
12	*S. aureus*	0.5	0.19	2	0.5	Hand			NDR
13	*S. aureus*	1	0.19	0.25	0.094	Thermometer			NDR
14	*S. aureus*	0.75	16^*∗*^	2	4^*∗*^	Thermometer			SDR
15	*S. aureus*	1	0.064	0.23	0.064	Surgical instruments	•	•	MRSA/MDR
16	*S. aureus*	1.5	0.094	0.016	0.125	Stethoscope			NDR
17	*S. aureus*	0.75	0.75	0.125	0.19	Consultation tables			NDR
18	*S. aureus*	8	8^*∗*^	2	0.5	Consultation tables	•	•	VISA/MRSA
19	*S. aureus*	6	16^*∗*^	38^*∗*^	0.047	Ultrasound table			VISA
20	*S. aureus*	2	0.094	0.023	0.064	Stethoscope			NDR
21	*S. aureus*	1.5	0.38	0.25	0.125	Consultation tables			NDR
22	CoNS	1	0.064	0.047	0.19	Dog´s cage		•	MRS
23	CoNS	0.75	0.064	0.023	0.064	Dog muzzle		•	MRS
24	CoNS	0.75	1	0.023	0.25	Surgical instruments	•	MRS	
25	CoNS	1	0.064	0.023	0.19	Dog´s cage		•	MRS

^*∗*^NDR: no drug resistance; SDR: single drug resistance; MDR: multidrug resistance; MRSA: methicillin-resistant *Staphylococcus aureus*; VISA: vancomycin-intermediate *Staphylococcus aureus*; VAN: vancomycin; CLI: clindamycin; GEN: gentamicin; CIP: ciprofloxacin.

## Data Availability

The data used to support the findings of this study are included within the article.
